# Renal Amyloidosis and Crohn Disease

**DOI:** 10.31486/toj.20.0129

**Published:** 2021

**Authors:** Julián E. Barahona-Correa, Samuel-David Morales, Rafael Andrade-Pérez, Albis Hani

**Affiliations:** ^1^ Department of Internal Medicine, School of Medicine, Pontificia Universidad Javeriana, Bogota, Colombia; ^2^ Department of Pathology, School of Medicine, Pontificia Universidad Javeriana – Hospital Universitario San Ignacio, Bogota, Colombia; ^3^ Department of Pathology, School of Medicine, Fundación Universitaria de Ciencias de la Salud – Hospital de San José, Bogota, Colombia; ^4^ Department of Pathology and Laboratories, Fundación Santa Fé de Bogotá, Bogota, Colombia; ^5^ School of Medicine, Universidad de los Andes, Bogota, Colombia; ^6^ School of Medicine, Universidad Nacional de Colombia, Bogota, Colombia; ^7^ Gastroenterology Unit, Hospital Universitario San Ignacio, Bogota, Colombia

**Keywords:** *Amyloidosis*, *Crohn disease*, *Latin America*, *renal insufficiency–chronic*, *tumor necrosis factor-alpha*

## Abstract

**Background:** Secondary amyloidosis, a rare complication of Crohn disease (CD), is triggered by persistent systemic inflammation. Kidney involvement is the most frequent manifestation and is often characterized by nephrotic syndrome and kidney failure. This complication usually appears in patients with long-standing disease and is associated with increased morbidity and mortality risk. Diagnosis is by microscopic amyloid observation of tissue biopsy, and when the diagnosis is confirmed, the therapeutic objective is disease activity control. Response assessment is challenging because of a lack of reliable biomarkers.

**Case Report:** A 56-year-old male with a long-standing history of CD treated with a tumor necrosis factor-α inhibitor presented with an acute elevation of creatinine in association with clinical and laboratory markers of nephrotic syndrome. Kidney biopsy revealed renal amyloidosis. After treatment adjustment, although a stable creatinine was achieved, the patient had persistent impaired glomerular filtration rate.

**Conclusion:** As a systemic chronic inflammatory disorder, CD may present multisystemic morbidity, for which increased awareness among gastroenterologists is warranted. Renal amyloidosis is an infrequent extraintestinal complication of CD that may lead to chronic kidney impairment. Although evidence-based treatment is lacking, disease activity control is pivotal for management.

## INTRODUCTION

Amyloidosis is a rare disease characterized by the extracellular deposition of an insoluble fibrillar material known as amyloid that can ultimately lead to organ dysfunction. Virtually any organ may be affected. Several types of amyloidosis have been described, both hereditary and acquired, with the acquired types being the most common.^1^

The most frequent type is amyloid light-chain (AL) amyloidosis, also known as wild-type transthyretin amyloidosis, that is related to plasma cell dyscrasia. AL amyloidosis occurs when plasma cells produce abnormal protein fibrils made up of monoclonal immunoglobulin light chains.^1^

The second most common type is serum amyloid A protein (AA) amyloidosis in which the fibrils arise from serum AA protein, an acute-phase reactant that is synthesized because of its upregulation by inflammatory cytokines and is associated with chronic inflammation.^2^ Several chronic inflammatory conditions are associated with this disease, such as chronic infections (eg, tuberculosis and osteomyelitis), rheumatic diseases (eg, systemic vasculitis, rheumatoid arthritis, axial spondyloarthritis, and autoinflammatory syndromes), neoplasia, and inflammatory bowel disease (IBD).^2,3^ Because the kidney is usually the first and major involved organ, patients are at increased risk of requiring renal replacement therapy. The treatment of amyloidosis is based on adequate control of the inflammatory disease activity.^1,2^

Amyloidosis associated with IBD is a rare condition, with an estimated prevalence of 0.5%.^4^ Among patients with IBD, Crohn disease (CD) is the entity most often related to AA amyloidosis, with a prevalence of 1%, suggesting that 1 of every 95 patients with CD may present with systemic amyloidosis.^4^ We present the case of a male with CD and renal amyloidosis. To the best of our knowledge, our case is the first reported from Colombia.

## CASE REPORT

**Figure 1.**
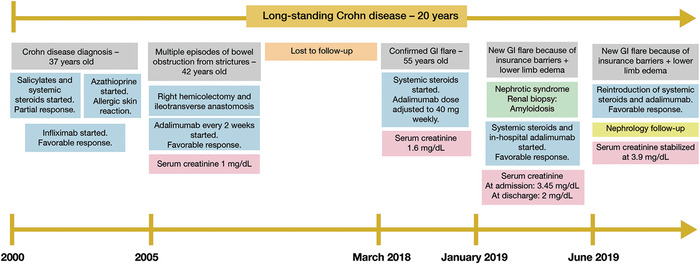
**Timeline of renal amyloidosis development in the presence of Crohn disease.** GI, gastrointestinal.

A 56-year-old male with a history of CD diagnosed when he was 37 years old presented to the emergency department with a gastrointestinal disease flare and lower limb grade 2 bilateral edema. When the patient was diagnosed with CD in 2000 (Figure 1), he was started on salicylates and systemic steroids and had only a partial response. Azathioprine was added but was discontinued shortly thereafter because of an allergic skin reaction. The patient was started on infliximab and had a favorable response.

Five years later while the patient was on infliximab, he presented with multiple episodes of bowel obstruction because of strictures that led to a right hemicolectomy and ileotransverse anastomosis. Biologic therapy was switched to adalimumab every other week, and disease control was achieved. The patient was lost to follow-up until March 2018, when he presented with a gastrointestinal disease flare that was confirmed by colonoscopy and histology reports. No infectious etiologies were observed. A systemic steroid (prednisone orally 1 mg/kg/day for 6 weeks with gradual tapering) was started, and the adalimumab (40 mg subcutaneous) injection interval was shortened to weekly. Adequate response was achieved when the patient received the weekly dose, but insurance issues presented barriers to access, and adalimumab was injected intermittently because of the insurance barriers.

**Figure 2.**
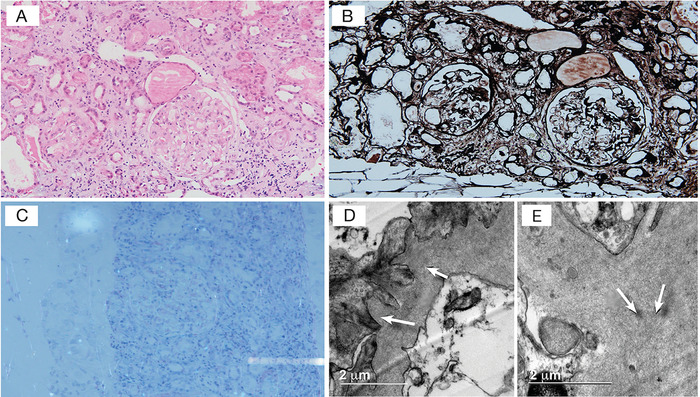
**Renal histopathology. (A) Hematoxylin and eosin stain shows a glomerulus with thickened and eosinophilic basal membranes with little obliteration of the capillary lumen, similar to findings in small arteries and arterioles. (B) Material does not stain in the argentic modified Jones stain; the membranes are predominantly clear with the silver stain. (C) Congo red stain under polarized light shows subtle apple green birefringence in the glomerular basal membranes. (D and E) Arrows in the ultrastructure point to organized fibril deposits consistent with amyloidosis**. (A color version of this figure is available at https://doi.org/10.31486/toj.20.0129.)

Ten months later (January 2019), the patient presented with a new disease flare (abdominal pain, increased bowel movements with nonbloody diarrhea up to 6 episodes per day) and lower limb edema. Admission creatinine was 3.45 mg/dL (creatinine at previous discharge was 1.6 mg/dL, and estimated glomerular filtration rate was 47 mL/min/1.72 m^2^ using the Chronic Kidney Disease Epidemiology Collaboration [CKD-EPI] equation). Prednisone 1 mg/kg/day and in-hospital adalimumab were started with a favorable response. Urinalysis showed proteinuria only, and urinary tract ultrasonography identified simple cysts, although no obstructive compromise or chronic disease changes were observed. Urine protein to creatinine ratio of 6,510 mg/g (reference, <300 mg/g) and hypoalbuminemia (1.6 g/dL; reference range, 4.2-5.5 g/dL) were present, thus nephrotic syndrome was considered. Histologic findings from renal biopsy were consistent with renal amyloidosis (Figure 2), which was considered secondary to CD chronic inflammation. Because of the patient's favorable response, the same treatment was continued, a serum creatinine of 2 mg/dL was achieved, and the patient was discharged home.

During follow-up in May 2019, the patient presented with an episode of dysphagia, and endoscopic findings were consistent with Kodsi stage 3 esophageal candidiasis and grade C peptic esophagitis. Treatment with fluconazole (200 mg/day orally for 14 days) and proton pump inhibitor therapy (esomeprazole 20 mg orally twice daily for 8 weeks) resulted in symptom resolution. No disease-associated compromise was observed.

In June 2019, the patient presented with a new disease flare for reasons similar to the May 2019 presentation and had an excellent response to medication reintroduction. Magnetic resonance enterography identified a 35-mm ileal stenosis at the ileotransverse anastomosis that was confirmed by colonoscopy. Edematous mucosa and multiple ulcers (8-10 mm) were noted at the ileotransverse anastomosis; at the ileum, 10 cm above the anastomosis, was an unenterable concentric stenosis. These endoscopic findings were consistent with a Rutgeerts score of i4, as diffuse inflammation with large ulcers and narrowing were present. No bowel obstruction was observed. After fluid resuscitation, creatinine stabilized at 3.9 mg/dL (estimated glomerular filtration rate was 16 mL/min/1.72 m^2^ using the CKD-EPI equation) and urinary output was adequate, but lower limb edema and hypoalbuminemia (1 g/dL) were still present. Nephrology follow-up is ongoing, and adequate medication access to guarantee adherence was finally achieved with the insurer. During follow-up, urinary output has been stable, and renal replacement therapy has not been necessary.

## DISCUSSION

Our case demonstrates extraintestinal morbidity resulting from chronic inflammatory disease activity in CD, illustrated by the rare manifestation of renal amyloidosis and, as a consequence, chronic kidney disease.

The prevalence of IBD varies among countries, although it is particularly high in many developed countries such as the United States, and the working-age population is widely affected.^5^ Our patient displayed the usual clinical features associated with amyloidosis and IBD. Males with IBD appear to be more affected with amyloidosis than females, with a 2:1 ratio, and this phenomenon usually occurs in long-standing disease, with a mean diagnostic interval between IBD diagnosis and amyloidosis onset of 13.7 years (95% CI 11.7%-15.6%; range, 0-42 years^4^); simultaneous diagnosis has also been described.^4^ Our patient presented with renal amyloidosis onset 20 years after his CD diagnosis.

A nationwide analysis in the United States showed that patients older than 50 years represented >80% of cases of systemic amyloidosis in IBD between 2004 and 2012.^6^ Ileocolonic involvement is the most frequently observed location of IBD compromise (54% of cases, CI 34%-75%).^4^ In contrast to our case, the most frequently reported manifestation is enteric fistula (34.8% of cases, 95% CI 9.9%-59.7%), followed by perianal disease (27.5% of cases, 95% CI 17.7%-37.3%) and stricture (27.3% of cases, 95% CI 13.1%-41.6%).^4^ Extraintestinal manifestations are frequent (52.3% of cases, 95% CI 45.1%-59.5%).^4,6^

Kidney compromise is the most frequently reported manifestation of systemic amyloidosis in IBD, principally expressed as kidney failure (70% of cases, 95% CI 53.2%-86.7%) associated with nephrotic syndrome (56%-100% of cases reported in the literature) as in our case.^4^ Some other locations of amyloid deposition include the heart, thyroid, duodenum, colon, rectum, adrenal glands, pancreas, spleen, and bone marrow, although frequency in these locations is lower compared to kidney compromise.^7-10^ The gold standard for diagnosis is the presence of amyloid deposition in histologic specimens, although a negative result does not exclude the presence of amyloidosis, as deposits may be patchy.^2^ The presence of apple green birefringence under cross-polarized light in a tissue biopsy stained with Congo red is diagnostic of amyloidosis,^2^ as observed in our case (Figure 2). Electron microscopy is a useful tool to corroborate the amyloid and exclude another type of deposit, and immunohistochemistry helps to determine the type of amyloid protein.^2^ Denis et al reported that amyloid deposition tends to involve all kidney compartments, which may play a role in the development of interstitial fibrosis and tubular atrophy leading to kidney failure^8^; this histologic pattern was seen in our patient (Figure 2).

In a study in Egypt, 24% of patients in an IBD cohort (n=896) in a 10-year follow-up developed impaired renal function. The most frequent presentation was nephrotic- (37%) or subnephrotic-range (17%) proteinuria. The most frequent findings on kidney biopsy were renal amyloidosis (26%), mesangial proliferation with immunoglobulin A deposits (16%), focal segmental glomerulosclerosis (15%), and crescentic glomerulonephritis (15%).^11^ Thus, renal amyloidosis should be considered in the differential diagnosis for patients with renal impairment in IBD, particularly when they present with rapidly progressive glomerulonephritis.

Amyloidosis is a rare extraintestinal complication of IBD, and CD is associated with a higher prevalence and risk than ulcerative colitis (UC). This phenomenon has been attributed to the wider extension of inflammation in CD compared to UC.^12,13^ Because of the low prevalence of systemic amyloidosis in IBD, most data come from descriptive studies (principally case series) that either pooled patients with IBD^6,7,11,14-17^ or reported amyloidosis in patients with CD.^8-10,18^ Therapeutic studies are lacking because of the rarity of systemic amyloidosis in IBD, and treatment is based on observational studies, expert recommendations, and extrapolation from the rheumatic diseases literature.^4^ Most of the experience from observational studies was pooled by Tosca Cuquerella et al.^4^ The primary objective is to control systemic inflammation leading to amyloid deposition. Thus, CD activity must be regulated, tissue amyloid deposition should be avoided, and established deposits should be reduced. Tumor necrosis factor (TNF)-α inhibitors, particularly infliximab, have been consistently reported as an effective therapy for amyloidosis in CD^19-22^; extrapolated evidence from rheumatic disease treatment supports the efficacy of TNF-α inhibitors, but they are associated with an increased risk of infection.^23^ Other strategies may be considered, including salicylates, methotrexate, and azathioprine; in addition, cyclosporine, mycophenolate, and thalidomide have been used.^2,8,10,11^ Colchicine has been proposed as an effective therapy, based on a favorable response of secondary amyloidosis in the context of periodic fever syndromes, such as familial Mediterranean fever, particularly during the prebiologics era.^2,4,7,10^ As these patients often present with nephrotic syndrome, the use of angiotensin-converting enzyme inhibitors, statins, and anticoagulation should be considered based on individual case assessment.^4^ In our patient, an effective therapy based on TNF-α inhibition (ie, adalimumab) was indicated. Nonetheless, insurance barriers undermined adequate adherence and probably a satisfactory response.

The presence of amyloidosis in patients with IBD is associated with an increased risk of hospitalization and mortality when compared to its absence. These patients appear to more often develop multisystem involvement, including cardiomyopathy.^4,6^ Although evidence to suggest biomarker monitoring is scarce, some authors have proposed that measuring serum amyloid A may be a useful strategy to assess amyloidosis progression, as C-reactive protein can be normal even when the disease is not responding.^14^

## CONCLUSION

As a systemic chronic inflammatory disorder, CD may present with multisystemic morbidity for which increased awareness among gastroenterologists is warranted, particularly for patients with long-standing disease. Renal amyloidosis is an infrequent extraintestinal complication of CD that is triggered by systemic inflammation, may lead to chronic kidney impairment, and appears to be associated with an increased morbimortality risk. Although evidence-based treatment is lacking, disease activity control is pivotal for management, and extrapolated evidence supports the use of TNF-α inhibitors as an effective therapy.

## Notes

*This article meets the Accreditation Council for Graduate Medical Education and the American Board of Medical Specialties Maintenance of Certification competencies for Patient Care and Medical Knowledge.*
